# Generation of Fel d 1 chain 2 genome-edited cats by CRISPR-Cas9 system

**DOI:** 10.1038/s41598-024-55464-0

**Published:** 2024-02-29

**Authors:** Sang Ryeul Lee, Kyung-Lim Lee, Seok-Hwan Song, Myeong-Don Joo, Seo-Hyun Lee, Ji-Su Kang, Seon-Min Kang, Muhammad Idrees, Jae-Wook Kim, Il-Keun Kong

**Affiliations:** 1 Animal, Dairy, and Veterinary Sciences Department, College of Agriculture and Applied Sciences, Utah State University, Logan, UT 84322 USA; 2 TheKingKong Corp. Ltd., Gyeongsang National University, Jinju, Gyeongnam Province 52828 Republic of Korea; 3 Division of Applied Life Science (BK21 Four), Gyeongsang National University, Jinju, Gyeongnam Province 52828 Republic of Korea; 4 Institute of Agriculture and Life Science, Gyeongsang National University, Jinju, Gyeongnam Province 52828 Republic of Korea; 5 Moores Cancer Center, University of California, San Diego, La Jolla, CA 92093 USA

**Keywords:** Allergen, Fel d 1, CH2, Genome-edited cats, CRISPR-Cas9, Hypoallergenic cats, Cloning, Genetic engineering, Biological models, Immunological models, Biotechnology

## Abstract

Allergens from domestic cats (*Felis catus*) cause allergy-related health problems worldwide. Fel d 1 is a major allergen that causes severe allergic reactions in humans, including rhinitis, conjunctivitis, and life-threatening asthma. Therefore, patients with cat allergies anticipate hypoallergenic cats. We successfully generated Fel d 1 chain 2 (CH2) genome-edited cats using the CRISPR-Cas9 system in this study. T7 endonuclease 1 assay and Sanger sequencing were used to confirm the mutation in CH2 genome-edited cats. Fel d 1 level in CH2 genome-edited cats were assessed by enzyme-linked immunosorbent assay (ELISA). Remarkably, ELISA showed that the level of Fel d 1 in the CH2 homozygous genome-edited cat (Name: Alsik) was extremely low compared with that in wild type domestic cats and could be hypoallergenic cats. Additionally, we successfully cloned the CH2 homozygous genome-edited cat using cytoplasm injection clone technology. The cloned CH2 homozygous genome-edited cat was verified using microsatellite analysis. Creating hypoallergenic cats using the CRISPR-Cas9 system is a significant step forward because these cats can safely approach allergic patients.

## Introduction

Cats (*Felis catus*) are among the most popular companion animals in the world^1^. However, patients allergic to cats are more likely to avoid having cats as companion animals because cat allergens are one of the most common sources of human allergies. In western countries, sensitivity to cat allergens is present in 10–30% of the general population^2,3^. However, there are currently no therapeutic methods other than the antihistamine medication^4^ that can be used. The World Health Organization/International Union of Immunological Societies (WHO/IUIS) allergen nomenclature lists cat allergens from Fel d 1 to Fel d 8^3^. In particular, Fel d 1, the major allergen, affects up to 90% of cat-allergic patients and accounts for 60–90% of the overall allergenic activity of cat dander^5,6^. Given that Fel d 1 is a very sticky protein, it can be challenging to remove it from contaminated carpets and clothes^7^. Additionally, as Fel d 1 is airborne, it can easily spread to areas without cats^8^. Fel d 1 is a 36 kDa heterotetrameric protein composed of two genes, Fel d 1 chain 1 (CH1) and Fel d 1 chain 2 (CH2), covalently linked via three disulfide bonds^9,10^. CH1 is an 8 kDa protein consisting of 70 amino acids (Supplementary Fig. 1a), while CH2 is a 10 kDa protein and has two dominant forms (Supplementary Fig. 1b)^9,10^. The long form consists of 92 amino acids, while the short form consists of 90 amino acids. The long form is mainly expressed in the salivary glands. The short form is preferentially expressed in the skin^9,10^. The physiological and biological functions of Fel d 1 have yet to be determined. However, there are reports on the similarity of Fel d 1 and rabbit uteroglobin^9^. Then several studies have noted the potential roles of Fel d 1 in immune regulation and epithelial defense because uteroglobin has functions in restricting the immune response against the embryo during implantation and protecting skin^9,11–13^. Many scientists have explored various methods to eradicate or limit the amount of Fel d 1 in cats, including conjugate vaccine containing recombinant Fel d 1^8^ or egg powder loaded with anti-Fel d 1 antibodies (Immunoglobulin Y; IgY)^13^. In addition, it was difficult to generate a hypoallergenic cat due to the slow development of the genome editing technique. Recently, the clustered, regularly interspaced short palindromic repeats (CRISPR)-Cas9 system has been developed and applied in many animals to induce genome editing. In 2022, Brackett et al. confirmed that the CRISPR-Cas9 system could eradicate Fel d 1 in feline cells with in vitro transfection study^14^. However, to date, there have been no reports on the development of hypoallergenic cats or the application of the CRISPR-Cas9 system to generate transgenic domestic cats. In this study, we report the first generation and cloning of CH2 genome-edited cats which can be hypoallergenic, using the CRISPR-Cas9 system.

## Results

### Preparation and in vitro verification on CH2 sgRNAs

To edit the CH2 genome, we designed single guide RNAs (sgRNAs) targeting the CH2 (Supplementary Fig. 2, Supplementary Table 1) using CHOPCHOP (https://chopchop.cbu.uib.no/) and CRISPR RGEN tools (rgenome.net) based on the CH2 sequence (Supplementary Fig. 3) in NCBI (https://www.ncbi.nlm.nih.gov). Before the sgRNAs were designed, we confirmed the CH2 sequence we would use since each cat could have a different genome sequence (Supplementary Fig. 3). Based on the CH2 sequence, we designed and purchased the sgRNA candidates for CH2, which contains two candidates (C2-1, C2-2) (Supplementary Table 1) from Integrated DNA Technologies (IDT, Coralville, IA, USA). Through in vitro validation, we confirmed that both candidates had high cleavage efficiency on the target regions (Supplementary Fig. 4, Supplementary Table 2). However, we selected C2-1 because C2-1 is closer to the start codon than CH2 (Supplementary Fig. 4, Supplementary Table 2). Afterward, we transcribed in vitro the mRNAs of C2-1 and Cas9 to be microinjected into the feline zygotes (Supplementary Table 2).

### Generation of CH2 genome-edited cats

We microinjected the mixture of C2-1 and Cas9 mRNA into the cytoplasm of cat one-cell stage embryos because it was very challenging to differentiate each pronucleus from the dark, lipid-filled cytoplasm in cat one-cell stage embryos (Supplementary Fig. 5). Following microinjection, twenty microinjected embryos were transferred into the oviducts of pseudopregnant recipient female cats (Supplementary Table 3). This represents the whole process related to microinjection and embryo transfer to generate CH2 genome-edited cats (Supplementary Fig. 6). On day 60, only one pseudopregnant recipient female cats gave birth to two kittens (Supplementary Fig. 7, Supplementary Table 4).

**Figure 1:**
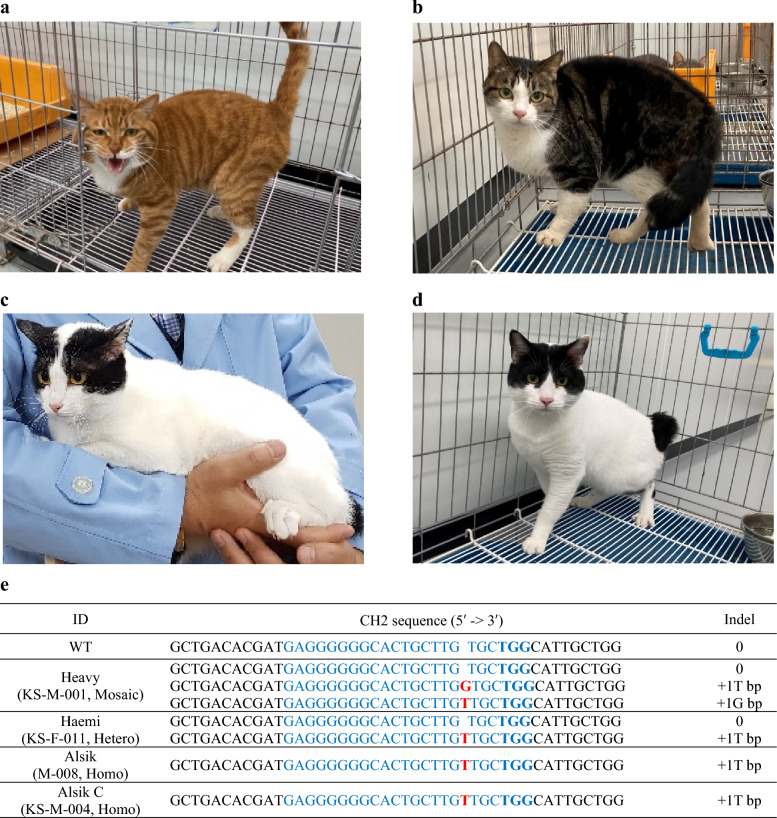

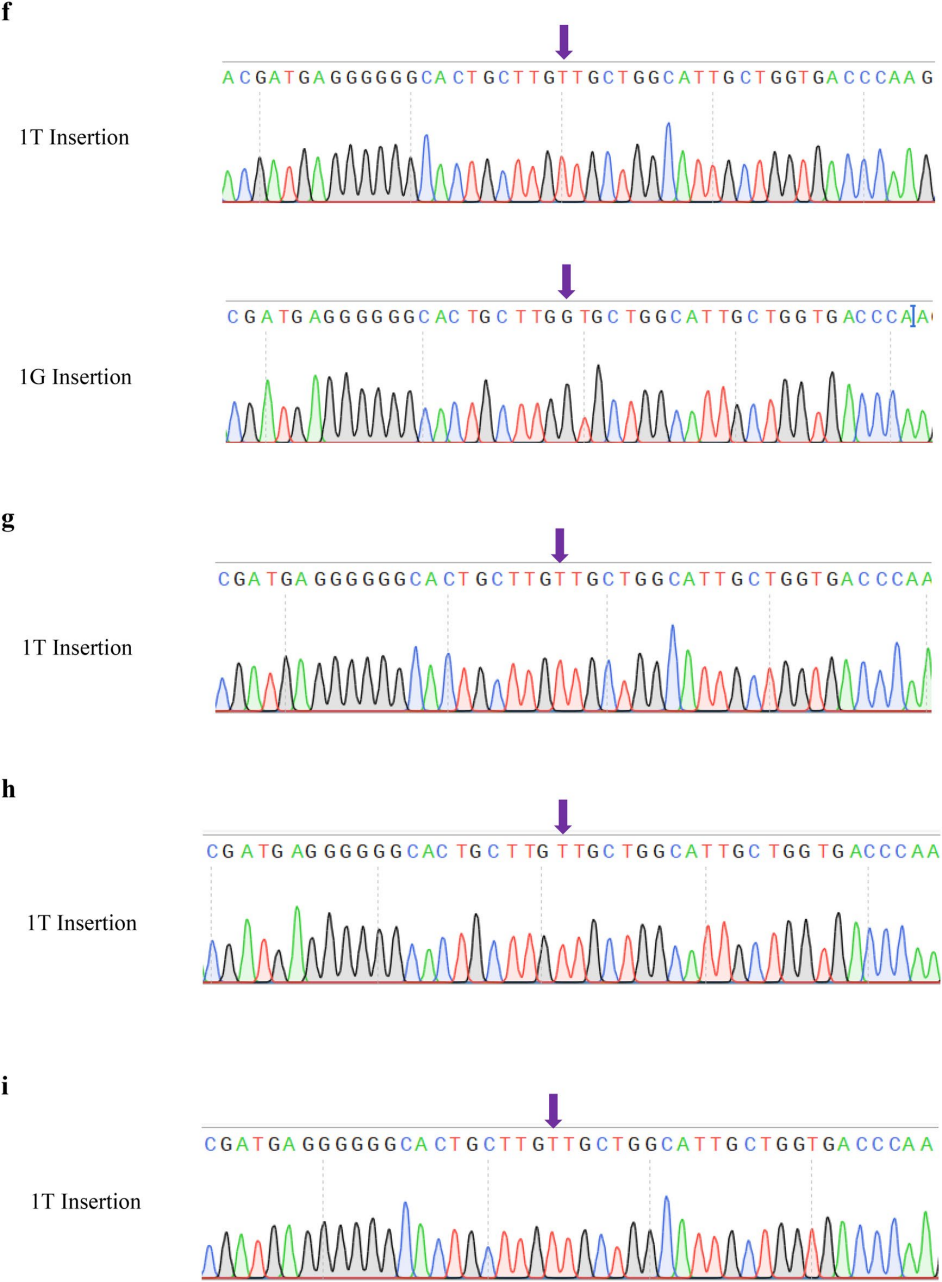
Generation and cloning of CH2 genome-edited cats using the CRISPR-Cas9 system and Sanger sequencing. (**a**) The CH2 genome-edited founder cat (Male, KS-M-001, Heavy). (**b**) The CH2 genome-edited founder cat (Female, KS-F-011, Haemi). (**c**) The CH2 genome-edited cat (F1, Male, CH2^−/−^, M-008, Alsik). (**d**) The CH2 genome-edited cloned cat (Male, CH2^-/-^, KS-M-004, Alsik C) from “Alsik”. (**e**) The modified target region in each CH2 genome-edited cat. (f) The sequencing chromatogram of the target region of the CH2 genome-edited founder cat (Heavy). (**g**) The sequencing chromatogram of the target region of the CH2 genome-edited founder cat (Haemi). (**h**) The sequencing chromatogram of the target region of the CH2 genome-edited cat (Alsik). (**i**) The sequencing chromatogram of the target region of the CH2 genome-edited cloned cat (Alsik C). “Heavy” and “Haemi” were generated by cytoplasmic microinjection of sgRNA C2-1 and Cas9 mRNA and embryo transfer. “Alsik” was generated from the mating of “Heavy” and “Haemi”. “Alsik C” was cloned from “Alsik” via CICT. Through the use of PCR and Sanger sequencing, mutations are found in every CH2 genome-edited cat. The blue highlights indicate the Cas9-targeted CH2 sequences. The PAM sequence is highlighted in bold blue. Bold red is used to highlight the indels. Each CH2 genome-edited cat’s DNA sequence is in alignment with the WT cat’s DNA sequence. CICT; cytoplasm injection cloning technology. ID; identification. CH2; Fel d 1 chain 2. Indel; insertion/deletion. PAM; protospacer adjacent motif. WT; wild type. Ns; nucleotides. T; thymine. G; guanine.

According to the T7 endonuclease 1 (T7E1) assay and Sanger sequencing with optimized primers (Supplementary Table 5), the first kitten (Male, Identification Number: KS-M-001, Name: Heavy) was mosaically mutated in the CH2 genome (Fig. 1a, Supplementary Figs. 7, 8). The second kitten (Female, Identification Number: KS-F-011, Name: Haemi) was heterozygous mutated in the CH2 genome (Fig. 1b, Supplementary Figs. 7, 8). We mated “Heavy” and “Haemi” as soon as they attained sexual maturity to produce CH2 homozygous genome-edited cats. “Haemi” delivered six new kittens (Supplementary Fig. 9). One of them (Male, Identification Number: M-008, Name: Alsik) was homozygous mutated in its CH2 genome (CH2^−/−^; Fig. 1c, Supplementary Fig. 10), and the other three cats (Males, Identification Number: M-003, M-004, and M-007) were heterozygously modified in their CH2 genome (CH2^+/−^; Supplementary Fig. 9).

### Cloning of CH2 genome-edited cat

We successfully cloned a CH2 homozygous genome-edited cat (Male, Identification Number: KS-M-004, CH2^−/−^, Name: Alsik C) from cat M-004 (Fig. 1d) using cytoplasm injection cloning technology (CICT)^15^. The cloned cat, Alsik C, revealed identical sequences of the CH2 genome with “Alsik” (Fig. 1e-i). Microsatellite analysis confirmed that the cloned “Alsik” and “Alsik C” were identical (Supplementary Table 6, 7).

### Enzyme-linked immunosorbent assay (ELISA) on CH2 genome-edited cats

**Figure 2:**
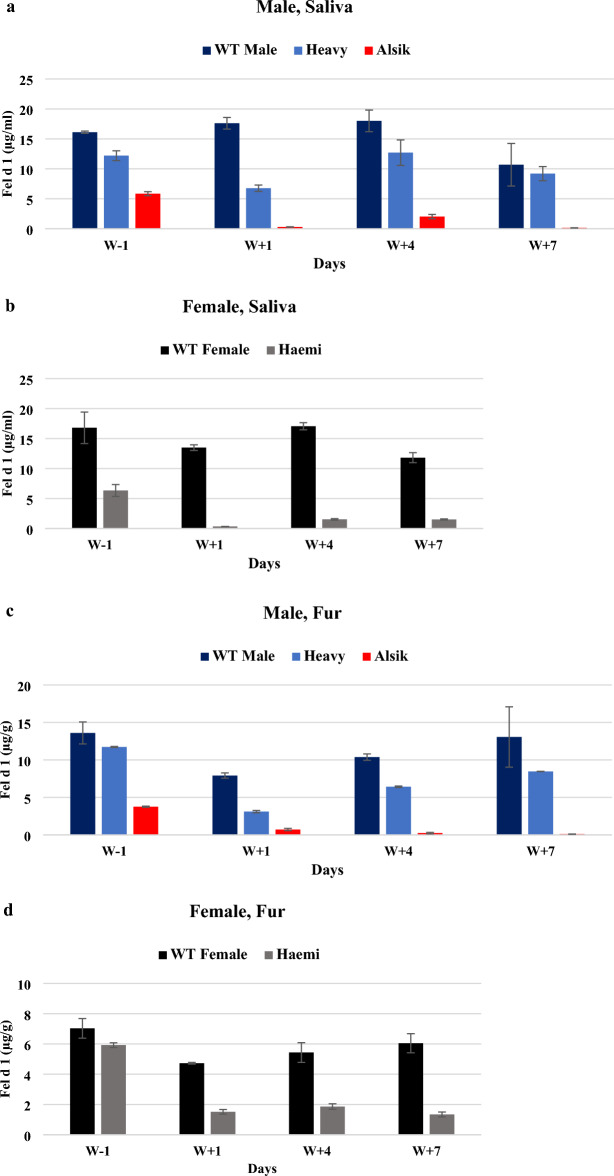
Production level of Fel d 1 in CH2 genome-edited cats before- and after washing. (**a**) Fel d 1 levels in the saliva of CH2 genome-edited male cats. (**b**) Fel d 1 levels in the saliva of CH2 genome-edited female cats. (**c**) Fel d 1 levels in the fur of CH2 genome-edited male cats. (**d**) Fel d 1 levels in the fur of CH2 genome-edited female cats. The production level of Fel d 1 was expressed as mean with standard deviation. The statistical significance of each value is presented in Supplementary Table 8. W; washing. W-1; the day before washing. W + 1; the day after washing. W + 4; day 4 after washing. W + 7; day 7 after washing.

ELISA was erformed to investigate the levels of Fel d 1 in saliva and fur from the two parent cats, “Heavy” and “Haemi” with their newborn kitten cat, “Alsik”, compared to wild type (WT) cats between the day (W − 1) before washing and the day (W + 1), the day 4 (W + 4), and the day 7 (W + 7) after washing, as soon as “Alsik” reached 6 months old. WT, “Heavy”, “Haemi”, and “Alsik” were individually housed in a regular animal room similar to the house owner’s house of a companion animal. When compared to the WT male cat, the saliva of “Alsik” on the day (W − 1) before washing had a statistically low level of Fel d 1 (5.86 µg/ml versus 16.11 µg/ml, *P* < 0.05, Fig. 2a, Supplementary Table 8). Compared to the day (W − 1) before washing, the majority (94.7%) of Fel d 1 in the saliva of “Alsik” was eliminated on the day (W + 1) after washing (Fig. 2a, Supplementary Table 8). Remarkably, on day 7 (W + 7) after washing, 98.6% of Fel d 1 in the saliva of “Alsik” was reduced (0.15 µg/ml vs. 10.69 µg/ml, *P* < 0.05), compared to the WT male cat (Fig. 2a, Supplementary Table 8). Compared with the day (W-1) before washing, 97.4% of Fel d 1 in the saliva of “Alsik” was removed on day 7 (W + 7) after washing (Fig. 2a, Supplementary Table 8). Alternatively, “Haemi” showed a drastically low level of Fel d 1 on day 7 (W + 7) after washing compared to the day (W − 1) before washing (1.51 µg/ml vs. 6.35 µg/ml) meaning that 76.2% of Fel d 1 was reduced (Fig. 2b, Supplementary Table 8). Eventually, the level of Fel d 1 in the saliva of “Haemi” was statistically low (1.51 µg/ml), compared with the WT female cat (11.81 µg/ml) on day 7 (W + 7) after washing (*P* < 0.05, Fig. 2b, Supplementary Table 8). Because of CH2 genome disruption, CH2 genome-edited cats have low levels of Fel d 1. However, the level of Fel d 1 in the saliva of WT male and female cat was slightly reduced or increased after washing (Fig. 2a,b, Supplementary Table 8), implying that the level of Fel d 1 in the WT female cat was unaffected by washing and that the level of Fel d 1 in the WT male cat was not significantly reduced by washing.

In fur, the level of Fel d 1 was significantly decreased by 72.4% in “Alsik” on the day (W − 1) before washing, compared with that in the WT male cat (3.75 µg/g versus 13.6 µg/g, *P* < 0.05, Fig. 2c, Supplementary Table 8). However, on day 7 (W + 7) after washing, the WT male cat recovered up to 96% of the Fel d 1 level in its fur compared to the day (W − 1) before washing (13.06 g/g versus 13.60 g/g). Especially, the level of Fel d 1 in the fur of “Alsik” was extremely low on day 7 (W + 7) after washing compared to the WT male cat, and up to 99.2% of the Fel d 1 had been eliminated (0.10 µg/g versus 13.06 µg/g, *P* < 0.05, Fig. 2c, Supplementary Table 8), similar to the level of Fel d 1 in the saliva. Moreover, the fur of “Haemi” also had a remarkably low level of Fel d 1, compared with the WT female cat, regardless of washing (Fig. 2d, Supplementary Table 8). This means that the hypoallergenic effect caused by CH2 alteration may affect females more frequently than males. Taken together, our data demonstrate that CH2 genome-edited founder cats, “Heavy”, “Haemi”, and CH2 homozygous genome-edited cat, “Alsik” showed low levels of Fel d 1. Especially, the CH2 homozygous genome-edited cat (Alsik) has an exceptionally low level of Fel d 1.

### Off-target analysis

We used polymerase chain reaction (PCR) and Sanger sequencing to analyze the putative off-target candidates to determine whether any off-target events happened in “Alsik” (Supplementary Table 9). The analysis confirmed that there were no off-target events by the CH2 sgRNA, C2-1 (Supplementary Figs. 11, 12).

## Discussion

Since they were first domesticated 9500 years ago^16^, cats have gained enormous popularity as companion animals worldwide. However, many people around the world have given up having cats as pets because of the allergens they produce. The primary genetic source of this allergic barrier is Fel d 1, which scientists worldwide are working to understand and treat as a remedy for the allergenic problems caused by Fel d 1 in cats, such as developing a vaccine against recombinant Fel d 1 and applying chicken IgY^17^. Additionally, Brackett et al.^14^ investigated the sequence of the conserved coding regions of the CH1 and CH2 genes from 50 domestic cats. Based on these sequences, they designed sgRNAs of CH1 and CH2 genes and analyzed the in vitro cleavage efficiency of CH1 and CH2 sgRNAs^14^. However, their report did not show the generation of the genome-edited cats, even though their report showed the possibility of applying the CRISPR-Cas9 system to cats. To date, there was still no noteworthy discovery regarding the blocking of Fel d 1. In this study, we focus on and describe the creation and cloning of a hypoallergenic cat with the world's first genome-edited using the CRISPR-Cas9 system on the CH2 genome locus of the Fel d 1. Also, sgRNAs used in their report differ from sgRNA (C2-1) used in this research.

The reproduction physiology of middle-sized animals, such as cats, and small-sized animals, such as mice and rats, differs from one another. The application of the mouse as a lab test animal model for the last three decades was based on its large number of eggs and short gestation period, as well as its whole genome sequencing analysis^18^. Until recently, there was no efficient genome editing technology to cause mutations in larger and polytocous animals. Since Cong et al.^19^ published the generation of the genome-edited mice using CRISPR-Cas9 system, the researchers have generated several genome-edited animals, such as cattle^20^, pigs^21^, sheep^22^, rabbits^23^, goats^23^, and chicken^24^. We then employed this technique to induce genome editing in Fel d 1 chain 2. However, research on genome-edited cats was difficult because of the challenge of recovering the cat's embryo sufficiently and the invisibility of the pronucleus inside the embryos (Supplementary Fig. 5).

We developed two CH2 genome-edited founder cats as depicted in Supplementary Fig. 7: one CH2 genome mosaically edited male cat (Heavy) and another CH2 genome heterozygously edited female cat (Haemi). Both cats are hypoallergenic founder cats because an ELISA test revealed that their Fel d 1 protein levels had dropped (Fig. 2, Supplementary Table 8) even though they were not homozygous on the mutated CH2 genome. The level of Fel d 1 in saliva and fur was significantly decreased by 61.6% and 70.9% in “Heavy” on the day (W + 1) after washing, compared with that in the WT male cat (6.77 µg/g vs. 17.61 µg/g, 3.1 µg/g versus 7.91 µg/g, *P* < 0.05, Fig. 2, Supplementary Table 8). However, on day 7 (W + 7) after washing, “Heavy” recovered up to 75.5% of the Fel d 1 level in its saliva compared to the day (W − 1) before washing (13.06 g/g vs. 13.60 g/g). Especially, “Haemi” showed a drastically dropped Fel d1 level in saliva (1.51 µg/g vs. 11.18 µg/g) and fur (1.34 µg/g vs. 6.05 µg/g) on day 7 (W + 7), compared with that in WT female cat (Fig. 2, Supplementary Table 8). When evaluating saliva and fur samples on day 7 post-washing, most of the Fel d 1 protein in the unique CH2 homozygous genome-edited cat (Alsik) that was created by mating male and female founder cats (Heavy and Haemi) was removed (Fig. 2, Supplementary Table 8). We estimate that the residual Fel d 1 concentration of the CH2 homozygous genome-edited cat (Alsik) would be from the environment because WT cats and CH2 homozygous genome-edited cats were housed in the same room even though they were individually caged. In addition, we may therefore infer that the CH2 homozygous genome-edited cat (Alsik) is a hypoallergenic cat.

In 1992, a study found that denatured Fel d 1 could not function as an allergen but could affect the conformational structure of Fel d 1, similar to alkylation and reduction^25^. In this study, we frameshifted the CH2 genome of CH2 genome-edited cats to disrupt the disulfide bond between CH1 and CH2 and, the ELISA results support this theory^25^. Saarne et al.^26^ hypothesized that breakage of disulfide bonds can reduce allergic reactions to human IgE. We estimated that the region immediately before the first cysteine residue (C24, Supplementary Fig. 1b), which was broken by C2-1, caused a disturbance in the CH2 genome. Our ELISA report supported the theory previously proposed by Saarne et al.^26^. Taken together, we believe that only the mutation in the CH2 genome locus can affect Fel d 1 expression and disrupt its secretion and that the mutation in CH2 disturbs Fel d 1 secretion. CH2 genome-edited cats we generated and cloned were very healthy and active, and there was no special issue with their health.

Owning to airborne contamination, Fel d 1 was present everywhere, even in the absence of cats. We measured the level of the Fel d 1 protein in the cat's saliva and fur since these were the primary exposures to the Fel d 1 protein. Some differences were detected in the level of Fel d 1 protein between saliva and fur samples during the experimental process, and the level of Fel d 1 protein in fur was stable and consistent, while in saliva, it was inconsistent and not completely influenced by the washing process. It seems that the interiors of their mouths were not wiped out because they were difficult to clean. As a result, the finding of Fel d 1 protein in saliva indicated considerable fluctuation in all cats. However, ELISA revealed that “Alsik” had reduced levels of Fel d l protein in saliva and fur.

Fel d 1 was contaminated through the air because WT cats and CH2 genome-edited cats were maintained individually and housed inside the same room and we used the washing process to remove Fel d 1 which originated from the environment. Saliva and fur were collected before washing to investigate the level of Fel d 1, which was from the environment as they were housed in the same room. The cats were naturally contaminated after washing, and the quantity of Fel d 1 in a regular animal room was polluted after washing. Then, after removing the relevance of Fel d 1 from the environment through the washing process, we collected saliva and fur from WT and CH2 genome-edited cats to investigate the level of Fel d 1. The cats underwent a minimum amount of stress while being showered and had every area of their bodies cleansed including their mouths during the washing process. To investigate the production levels of Fel d 1 under the same housing conditions and to avoid contact with each other, we individually housed WT cats and CH2 genome-edited cats in a regular animal room similar to the house owner’s house of a companion animal. Consequently, even though it was maintained in a typical home, it was not significantly poisoned (Fig. 2, Supplementary Table 8).

Usually, the level of Fel d 1 was fully recovered on the day (W + 1) after washing in domestic cats^27^. In our results, the level of Fel d 1 in CH2 genome-edited cats was extremely low, whereas the level of Fel d 1 in domestic WT cats recovered to some level on the day (W + 1) after washing even if they were in a regular room (Fig. 2, Supplementary Table 8). Additionally, we investigated the level of Fel d 1 by day 7 (W + 7) after washing to find the level of Fel d 1 of CH2 genome-edited cats affected by the air contamination in a regular room after washing. Our results show that the level of Fel d 1 of CH2 genome-edited cats is not affected by the air contamination even though they were in a regular room.

The likelihood of off-target events when creating transgenic or genetically modified using the CRISPR-Cas9 system is a subject of ongoing debate. In this study, four anticipated off-target candidates were used to assess the probability of off-target events on CH2 homozygous genome-edited cat, Alsik (Supplementary Figs. 11, 12). As a result, there were no off-target occurrences in the hypoallergenic cats in this study.

The production of Fel d 1 or allergens has been reported to be reduced in various cat breeds, including Balinese, Javanese, Siberian, Devon, Russian Blue, Cornish Rex, Bengal, Oriental Shorthair, LaPerm, and Sphynx^28^. These cats are disputed despite their reputation as less allergic cats. Since the generation of Fel d 1 nearly completely stops at day 4–7 after washing, CH2 homozygous genome-edited cat (Alsik) that we developed can be the hypoallergenic cat in comparison to these cats (Fig. 2, Supplementary Table 8). Thus, the CH2 homozygous genome-edited cat that can live safely with its owner was successfully cloned.

Additionally, these Fel d 1 chain 2 genome-editied cats were not validated yet, thus should be validated as “hypoallergenic” in future studies. Especially, more clinical research on atopic patients using cats with the CH2 genome edited is required to examine the expression of IgE that triggers allergic reactions. To understand the biochemical makeup of Fel d 1 and the mechanism causing the release of cat allergens, we also need to expand our inquiry into the production of CH1 or CH1/CH2 genome-edited cats.

## Conclusions

To date, there have been no reports of genetically modified cats on the genome loci of Fel d 1, which is a major allergen. This study is the first to demonstrate that the CH2 genome in cats can be genetically engineered using the CRISPR-Cas9 system and cytoplasmic microinjection, and the CH2 genome-edited cat can be cloned by CICT. The CH2 homozygous genome-edited cat (Alsik) could be considered a hypoallergenic cat because it produces an exceptionally low level of Fel d 1 and can serve as a companion animal and potential therapeutic animal for those with an allergic reaction to Fel d 1. Further studies should focus on the generation of CH1/CH2 genome-edited cats and their clinical testing. Many people who suffer from cat allergens may find hope in this report. These advances have broadened the value of cats as therapeutic and disease animal models.

## Methods

### Chemicals

Unless otherwise described, all the chemicals were purchased from Sigma-Aldrich (St. Louis, MO, USA).

### Animals

The domestic recipient queens and the domestic donor queens were individually housed in stainless steel cages measuring 1.86 m × 0.73 m × 0.65 m (W × D × H) under the provision of dry food and water *ad libitu*^15^. The controlled environment, which was maintained at a temperature of 21–25 °C, underwent light cycling under a photoperiod of 14D:10N^15^. Gyeongsang National University (GNU, Republic of Korea) and the Institutional Animal Care and Use Committee (IACUC) approved all surgical techniques used (IACUC Protocol Number: GNU-170727-T0034) and the experimental procedures and methods were performed in accordance with ARRIVE guidelines (https://arriveguidelines.org). All of the offspring in our cat colony have been treated the vaccinations (Nobivac Feline 1-HCPCh + FeLV, MSD Animal Health, Rahway, NJ, USA) eight weeks after birth, apart from three weeks later, for feline rhinotracheitis, calici, panleukopenia, feline chlamydophila, and feline leukemia virus (FeLV). The queens for this experiment were selected carefully without any disease symptoms and then used for donor or recipient queens^15^. Our facility administered vaccinations (Felocell 4, Zoetis KR, Seoul, Republic of Korea) 12 weeks after birth, apart from four weeks later against feline herpesvirus, calicivirus, panleukopenia virus, and Chlamydia psittaci (Felocell 4, Pfizer, New York, NY, USA)^15^. For the domestic cats used during the study, we can confirm that they were obtained from the cat farm specifically built for research purposes. We obtained approval from the Institutional Animal Care and Use Committee (IACUC) to use these cats for our study. As these cats were not owned by individuals, there was no requirement to obtain consent from any owners.

### Superovulation and oocyte collection

The donor queens were a group of five adult cats that ranged in age from two to four years old and weigh between two and four kilograms (kg). The modified Yin et al. technique ensured ovarian follicular development of the donor cats^29^. In summary, 100 IU of human Chorionic Gonadotropin (hCG; Daesung, Republic of Korea) was administered 4 days after the injection of 200 IU equine Chorionic Gonadotropin (eCG; Daesung, Republic of Korea) into the donor cats at the Gyeongsang National University Animal Medical Center. We performed laparotomy after 24 h to expose the ovaries. Under general anesthesia with alfaxalone (alfaxan multidose; 4 mg/kg; Jurox, New South Wales, Autralia) and Tramadol (2 mg/kg; Jurox, New South Wales, Australia), the immature oocytes were aspirated from the ovaries using an 18 G needle (305,196, BD, Franklin Lakes, NJ, USA) attached to a 1 ml syringe (319,461, BD). We cultured the collected immature oocytes in vitro maturation (IVM) medium consisting of TCM199 (M7528) supplemented with 10% fetal bovine serum (FBS; 16,000–044, Gibco, Grand Island, NY, USA), 1 μg/ml estradiol-17β (E2758), 10 μg/ml follicle-stimulating hormone (FSH; HOR-285, Prospec, Israel), 0.6 mM cysteine (C7352), 0.2 mM sodium pyruvate (11,360–070, Gibco), and 1% penicillin/streptomycin (P4333) at 38.5 °C in an atmosphere of 5% CO_2_ for 4 h.

### Semen collection and cryopreservation

We collected cat sperm using artificial virginal methods^30^. In brief, the estrus of a teaser queen was induced by injecting 100 IU eCG. The recovered semen was then cleaned with D-PBS (14190–250, Gibco), centrifuged at 200×*g* for 5 min, and frozen following a cryopreservation protocol^30^. Extender I (Ext I) was created by adding 20% chicken egg yolk and 1% penicillin/streptomycin (P4333) to Tris buffer. Whereas, extender II (Ext II) was prepared by adding Ext I to 8% glycerol (G2025) and 1.0% Equex STM (Nova Chemicals, Calgary, Canada). The diluted semen with Ext I was placed in a 10 ml tube (SPL Life Sciences, Republic of Korea) and slowly cooled to 5 °C under 150 ml warm water immersion at 37 °C in a refrigerator for at least 2 h. Then, it was diluted with Ext II for at least 1 h. The cooled semen was packaged in a 0.25 ml straw (IMV Technologies, France) and kept at 5 cm above liquid nitrogen vapor before being submerged for long-term preservation in a cold chamber (5 °C).

### In vitro fertilization (IVF) and in vitro culture (IVC)

The post-thawed sperm was diluted in D-PBS and centrifuged at 200×*g* for 5 min. After extracting the supernatant and diluting the pelleted sperm with 500 μl of 20 μg/ml heparin (H3149) for 15 min, sperm capacitation was induced. In vitro fertilization of mature oocytes was performed for 6 h with 1–2 × 10^6^ cells/ml of capacitated sperm. We cultured the presumed zygotes in a 50 μl drop in vitro culture (IVC)-1 medium consisting of Charles Rosenkrans-Amino Acids (CR1-aa) medium^31^ supplemented with 44 μg/ml sodium pyruvate (11360–070, Gibco), 14.6 μg/ml L-glutamine (35030–081, Gibco), 10 μl/ml penicillin/streptomycin (P4333), 3 mg/ml bovine serum albumin (BSA: A6003), and 310 μg/ml glutathione (G6013) for 3 days, and then in IVC-2 medium for 7 days. The latter was prepared by replacing BSA (A6003) with 10% FBS (16000–044, Gibco) in the IVC-1 medium. For IVM, IVF, IVC-1, and -2, oocytes and embryos were grown in a 5% CO_2_ incubator at 38.5 °C with maximum humidity.

### The design of CH2 sgRNAs

Because of the critical variations in the CH2 sequence depending on the species, the CH2 sequence of the cat we used as an oocyte donor cat was PCR amplified and analyzed by Macrogen (Republic of Korea) (Supplementary Table 5), as aligned and confirmed with CH2 (NCBI accession number: X62478) in NCBI Gene database (https://www.ncbi.nlm.nih.gov/gene/). Based on the CH2 sequence of the domestic cat studied, sgRNA candidates next to the protospacer adjacent motif (PAM) sequence were designed using CHOPCHOP (https://chopchop.cbu.uib.no/)^32^ and CRISPR RGEN tools (rgenome.net)^33^ (Supplementary Fig. 2, Supplementary Table 1) to introduce the frameshift behind the start codon of CH2.

### Preparation of CH2 sgRNAs and Cas9

pX330-U6-Chimeric_BB-CBh-hSpCas9 plasmid (pX330, Addgene plasmid number 42230, 8484 bp) was cut with BbsI (R0539S, New England Biolabs, Ipswich, MA, USA) and confirmed by 1% agarose gel electrophoresis and gel purification. We annealed and ligated the oligonucleotides of CH2 sgRNAs to the linearized vector pX330 using T4 DNA ligase (M0202S, New England Biolabs). The ligated pX330-CH2 plasmids were then transformed and purified using a Qiagen miniprep kit (27,106, Qiagen, Valencia, CA, USA).

### In vitro validation of sgRNAs

We performed an in vitro validation for CH2 sgRNA candidates using the protocol Mehravar et al. used^34^. We prepared the PCR product with optimized primers (Supplementary Table 5) for in vitro validation. PCR was carried out using Phusion high-fidelity DNA polymerase (F530L, Thermo Fisher Scientific, Waltham, MA, USA) and optimized primers to detect the target region of CH2 (Supplementary Table 5). The purity of the PCR product was observed using SYBR nucleic acid gel stain (S7580, Thermo Fisher Scientific) following 2% agarose gel electrophoresis. The EnGen Spy Cas9 NLS Cas9 protein was purchased from New England Biolabs. Briefly, 30 nM sgRNA (IDT), 10X New England Biolabs buffer r3.1 (New England Biolabs), and 1 μM EnGen Spy Cas9 NLS Cas9 protein (New England Biolabs) were mixed and incubated at 25 °C for 10 min. The PCR product was then added and incubated at 37 °C for 1 h. Following incubation, 1 μl proteinase K (20 mg/ml, 9034, Takara, Japan) was added and incubated at 65 °C for 10 min. The incubated reactions were visualized by 2% agarose gel electrophoresis and the validity was analyzed using the ImageJ program^35^.

### In vitro transcription

The mRNAs for C2-1 were created in vitro, with Cas9, for microinjection into the cytoplasm of the one-cell stage embryos. We amplified the DNA template for Cas9 mRNA synthesis after linearization with XbaI and the DNA template for the transcription of C2-1 in vitro by PCR using primers flanked by the T7 promoter region (Supplementary Table 2). The PCR products were observed by agarose gel electrophoresis and purified by using a Qiagen PCR Cleanup Kit (28104, Qiagen). The C2-1 and Cas9 mRNA were then transcribed in vitro using the transcription synthesis kits MEGAshortscript T7 (AM1334, Ambion, Austin, TX, USA) and mMESSAGE mMACHINE mRNA (AM1344, Ambion), respectively. Both the synthesized C2-1 and Cas9 mRNAs were purified using the MEGAclear kit (AM1908, Ambion).

### Microinjection of CH2 sgRNA and Cas9 mRNA in cat zygotes

The presumed zygotes were washed with TCM199 supplemented with 0.003 μg/ml BSA and placed on the warm plate of the microscope system. The operating pipettes were set up under a microscope before the zygotes were placed, and the embryos were arranged using a holding pipette at an angle of 180° toward the polar body at the 6 or 12 of the clock. A microinjection technique (Femtojet system, Eppendorf, Germany) was used to inject a mixture of C2-1 and Cas9 mRNAs into the cytoplasm of zygotes. To generate CH2 genome-edited cats, microinjected zygotes were grown in IVC-1 medium in a 5% CO_2_ incubator at 38.5 °C with maximum humidity.

### The synchronization of the estrous cycle of the recipient queens and embryo transfer

As previously described, the microinjected embryos were implanted into the oviducts of recipient estrous-synchronized queens in the operating theater room using a 37 °C warm water vacuum bottle^36^. The estrous cycle stage was synchronized by injecting 200 IU eCG and 100 IU hCG, 4 days apart. Before surgery, we pre-medicated the recipient cats with medetomidine (10 μg/kg; subcutaneous injection; Domitor, Orion Pharma, Finland), atropine (0.04 mg/kg; subcutaneous injection; Jeil Pharmaceutical Co., Republic of Korea), acepromazine (0.1 mg/kg; subcutaneous injection; Samu Med., Republic of Korea), and cefazolin as antibiotic (25 mg/kg; intravenous injection; Chong Kun Dang Pharm Co., Republic of Korea). The cats were anesthetized using intravenous administration of 2 mg/kg etomidate (B. Braun Melsungen AG, Germany). Anesthesia was maintained by the exposure to 2.5% isoflurane (1,040,603, Butler Schein Animal Health, Visalia, CA, USA), along with oxygen, through an endotracheal tube. Throughout the surgery, saline solution was administered intravenously at 10 ml/kg/h. To prepare a column, we loaded the microinjected embryos into a catheter with approximately 0.5 ml of IVC-1 medium. Then, twenty microinjected embryos were surgically implanted into each estrous-synchronized recipient cat. After embryo implantation, the receipient cats were cared for in a separate clean room until their natural birth.

### T7E1 assay and Sanger sequencing

A Puregene Core Kit (158,445, Qiagen) was used to isolate genomic DNA from the cat ears. Genomic fragments containing C2-1 target regions were amplified from the genomic DNA by PCR using the optimized primers (Supplementary Table 5). The PCR products were visualized on a 2% agarose gel stained with SYBR nucleic acid gel stain and then applied to T7E1 assay (M0302L, New England Biolabs) where it was sequenced, cloned into the pcDNA3.1 cloning vector (V79020, Invitrogen, Waltham, MA, USA), and transformed into 5-alpha competent *E. coli* (C2987H, New England Biolabs). After spreading and overnight culture, 20 random colonies were collected and grown, and the plasmid DNA was isolated and sequenced. We confirmed the mutations by aligning the sequenced alleles with WT alleles.

### Washing and sampling

Saliva and fur were collected from each cat 24 h before washing as follows: saliva was collected with a micropipette and fur with grooming gloves. Following the veterinarian’s instructions, we washed the WT and CH2 genome-edited cats with a shampoo (HYPONIC Hypoallergenic Shampoo, Koonaent, Seoul, Republic of Korea) specific for cats, rinsed them with warm water, air-dried them to remove Fel d 1 from environmental conditions^37^, and investigated the expression of Fel d 1 in CH2 genome-edited cats. Subsequently, WT and CH2 genome-edited cats were transferred to a regular animal room and individually housed inside a regular animal room. The saliva and fur were then collected at 24, 96, and 168 h after washing to investigate the production of Fel d 1 which resulted from Fel d 1 genome-edited cats after removing Fel d 1 from the environment.

### Enzyme-linked immunosorbent assay (ELISA) analysis

We investigated the production level of Fel d 1 using the Fel d 1 ELISA 2.0 kit-Single Plate (EPC-FD1-1, Indoor Biotechnologies, Charlottesville, VA, USA), following the manufacturer’s protocol. We collected 1 mg of fur and 30 μl saliva in 3 ml water. Similarly, we induced the Fel d 1 secretion for 24 h at room temperature. The dilution factor for ELISA is 1/10, optimized to accurately detect Fel d 1. We analyzed the standard curve via online curve fitting (mycurvefit.com) in MyAssays (myassays.com). We then calculated and analyzed the concentrations from symmetrical sigmoidal calibrators derived from ELISA results using the four-parameter logistic (4PL) curve fit in Myassays.

### CICT

The nuclear donor cells were prepared from the ear tissue of the CH2 homozygous genome-edited cat (Alsik) and were then immersed in cell culture medium (Dulbecco's modified Eagle's medium, DMEM, 11965, Gibco) supplemented with 15% FBS, 1% L-glutamine, 1% non-essential amino acids (11140–050, Gibco), 1% penicillin/streptomycin^15^. Domestic queens weighing 2–4 kg each provided the recipient embryos. CICT was performed as described previously^15^. In brief, denuded mature oocytes were enucleated by removing the first polar bodies and adjacent ooplasm containing metaphase II stage chromosomes. The prepared nuclear donor cells were immersed in Sendai Virus (SV; Cosmo Bio, Japan)^38^, with approximately 30% of the cytoplasm derived from donor oocytes, and were co-injected into the perivitelline space of the enucleated recipient oocytes to restore the cytoplasmic volume that occurred before oocyte enucleation^39^. The reconstructed oocytes were fused via SV-mediated fusion and then incubated in a modified synthetic oviduct fluid medium for 2 h^40^. Reconstructed oocytes (cloned embryos) were activated in 5 M ionomycin (I3909) for 5 min and then incubated in 2 mM 6-dimethylaminopurine (6-DMAP; D2629) under humidified conditions with 5% CO_2_ at 38.5 °C for 4 h. When the time was correct, we implanted 4–15 cloned embryos into the oviducts of estrus-synchronized queens. Then, we took care of the receipient cats in a separate clean room until their natural birth.

### Microsatellite analysis of the cloned cats

We adhered to the parentage analysis procedure described by Yin et al.^29^ Nine feline DNA microsatellite markers (FCA229, FCA290, FCA305, FCA441, FCA078, FCA201, FCA224, FCA170, and FCA304) were used to identify the donor CH2 homozygous genome-edited cat (Alsik) and the cloned CH2 homozygous genome-edited cat (Alsik C)^41^. To analyze the microsatellite markers, we used genomic DNAs from the donor CH2 homozygous genome-edited cat (Alsik), cloned CH2 homozygous genome-edited cat (Alsik C), and WT cat, and obtained their primers from Macrogen (Supplementary Table 6). Noting that each forward primer was labeled with 6-FAM, a 3130xl Genetic Analyzer (Applied Biosystems, Waltham, MA, USA) was used to evaluate all PCR results, and the GeneScan Version 5 (Applied Biosystems) was used to calculate the allele sizes.

### Off-target analysis

Using CHOPCHOP or CRISPR RGEN tools, we estimated the likelihood of off-target regions from other chromosomes, except for Fel d 1 gene family in the cat genome. As a result, we evaluated four putative off-target candidates (Supplementary Table 9), using PCR and Sanger sequencing.

### Statistical analysis

Data are presented as mean values with standard deviations. One-way ANOVA was used to test statistical significance. Statistical significance was set at a *P* value of 0.05. The ELISA experiment and sampling was independently replicated at least twice. All statistical analyses were performed using SPSS version 29 (IBM SPSS Inc., Chicago, IL, USA).

### Ethical approval

The animals and procedures used in this study were in accordance with the guidelines and approval of the Institutional Animal Care and Use Committees (IACUC) at Gyeongsang National University (GNU).

### Supplementary Information

Supplementary Information.

## Data Availability

Data are contained within the article. The datasets generated and/or analysed during the current study are available in GenBank accession number, OQ943594 and NCBI, https://www.ncbi.nlm.nih.gov/gene/677879.
