# Supplementary material for: Generation of Fel d 1 chain 2 genome-edited cats by CRISPR-Cas9 system
Source: Sci Rep. 2024 Feb 29;14:4987. doi: 10.1038/s41598-024-55464-0 (PMC10904870; doi:10.1038/s41598-024-55464-0)
Supplement: Supplementary file 1 — Supplementary Information. [file 41598_2024_55464_MOESM1_ESM.docx]

## **Generation of Fel d 1 chain 2 genome-edited cats by CRISPR-Cas9 system**

**Sang Ryeul Lee^1,5,6^, Kyung-Lim Lee^2,6^, Seok-Hwan Song^2^, Myeong-Don Joo^3^, Seo-Hyun Lee^3^, Ji-Su Kang^3^, Seon-Min Kang^3^, Muhammad Idrees^3^, Jae-Wook Kim^2^ & Il-Keun Kong^2,3,4,*^**

**Supplementary Figures**

**Supplementary Figure 1.** mRNA and amino acid sequences of CH1 and CH2. (**a**) The mRNA and amino acid sequences of CH1. (**b**) The mRNA and amino acid sequences of CH2**.** Red was used to identify the start codon, and blue was used to identify the stop codon. Bold red was used to identify cysteines that form disulfide bonds. The underline indicates that the T-cell epitope binding location is represented by underlining. The amino acid sequence is at the bottom, and the mRNA sequence is at the top.

Abbreviations: CH1, Fel d 1 chain 1; CH2, Fel d 1 chain 2;

**a**

AUG UUA GAC GCA GCC CUC CCA CCC UGC CCU ACU GUU GCG GCC ACA GCA GAU UGU GAA AUU

M L D A A L P P C P T V A A T A D C E I

**UGC** CCA GCC GUG AAG AGG GAU GUU GAC CUA UUC CUG ACG GGA ACC CCC GAC GAA UAU GUU

**C** P A V K R D V D L F L T G T P D E Y V

GAG CAA GUG GCA CAA UAC AAA GCA CUA CCU GUA GUA UUG GAA AAU GCC AGA AUA CUG AAG

E Q V A Q Y K A L P V V L E N A R I L K

AAC **UGC** GUU GAU GCA AAA AUG ACA GAA GAG GAU AAG GAG AAU GCU CUC AGC UUG CUG GAC

N **C** V D A K M T E E D K E N A L S L L D

AAA AUA UAC ACA AGU CCU CUG **UGU** UAA

K I Y T S P L C

**b**

AUG AGG GGG GCA CUG CUU GUG CUG GCA UUG CUG GUG ACC CAA GCG CUG GGC GUC AAG AUG

M R G A L L V L A L L V T Q A L G V K M

GCG GAA ACU **UGC** CCC AUU UUU UAU GAC GUC UUU UUU GCG GUG GCC AAU GGA AAU GAA

A E T **C** P I F Y D V F F A V A N G N E

UUA CUG UUG GAC UUG UCC CUC ACA AAA GUC AAU GCU ACU GAA CCA GAG AGA ACA GCC

L L L D L S L T K V N A T E P E R T A

AUG AAA AAA AUC CAG GAU **UGC** UAC GUG GAG AAC GGA CUC AUA UCC AGG GUC UUG GAU

M K K I Q D **C** Y V E N G L I S R V L D

GGA CUA GUC AUG ACA ACC AUC AGC UCC AGC AAA GAU **UGC** AUG GGU GAA GCA GUU CAG

G L V M T T I S S S K D **C** M G E A V Q

AAC ACC GUA GAA GAU CUC AAG CUG AAC ACU UUG GGG AGA UGA

N T V E D L K L N T L G R

**Supplementary Figure 2.** Location of sgRNA candidates on the CH2 genome. Red pentagons indicate CH2 exons. The blue arrows indicate the location of the sgRNA candidates for CH2.

**
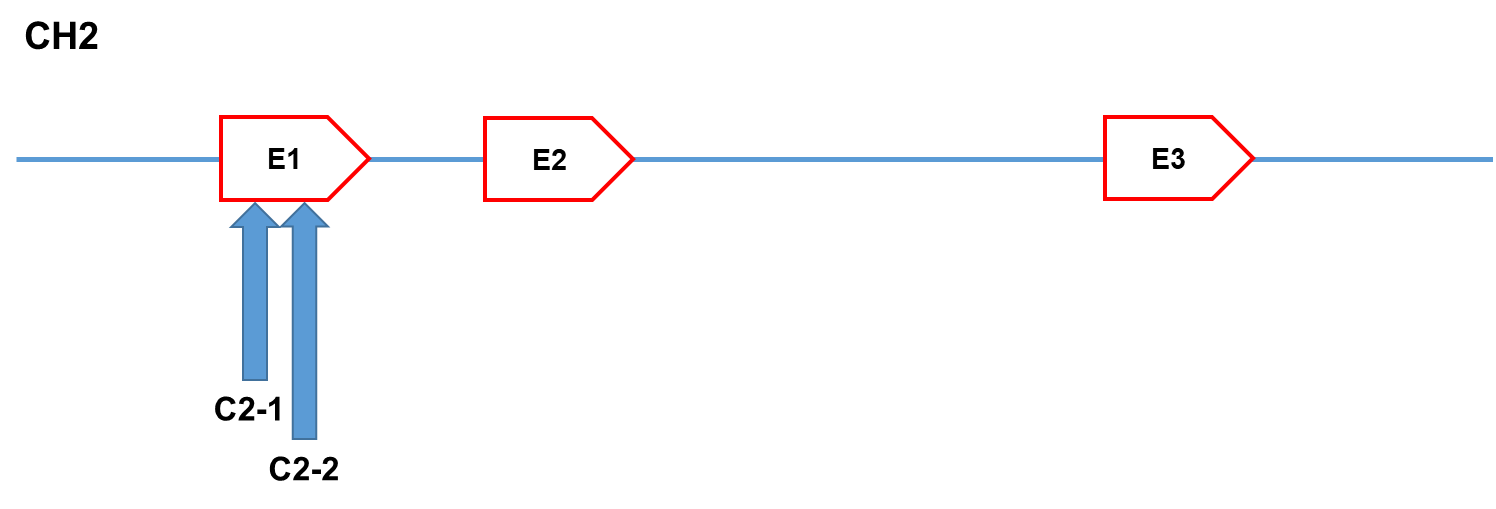
**Abbreviations: CH2, Fel d 1 chain 2; sgRNA, single-guide RNA; E, exon; C2-1, C2-2; candidate.

**Supplementary Figure 3.** The DNA sequence of the CH2 genome. The genomic DNA sequence of CH2. The start codon is highlighted in red, and the stop codon is highlighted in blue. sgRNA; single guide RNA. PAM; protospacer adjacent motif.

5’ CACATCCTCTCCAAGAGCTTTGTCCTCAAGAGTAGAAGGGCTTCCCACTCTTAACAGCCAAGGGTTGAGGAGCCACCCACATGTGCCAGGTCCCTGCCCACAGGCCTTTGGAGCTTCTGGCGGGGGGGGGGTGTGTGGGCTGGGCTTAGGGTGCTAGTAGTTTATAAAGCAGCAGAAATCCTGTCCTGAGCAGAGCATTCTAGCAGCTGACACG**ATG**AGGGGGGCACTGCTTGTGCTGGCATTGCTGGTGACCCAAGCGCTGGGCGTCAAGATGGGTGAGAGCAGATGGAGGGACAGAGGACCTTCCTGATCCTTGCCCTGCTCTATCTCACTCCTTCACCTCCCATGGTGATCTCCAAACAGGTTCTAGCCACAAAGTTAAGCGGCCATGGGGAGATCATTGTCCAGGAGTCCTGCAGAACCCCCCTGATGTTTTTAGTCGTTGAATGGAGGGAGAGGTTTGGAGATGGAGGGGTCATTAGTCGTGCACACAATAGGGGAGAGTTAGTTGGGGGTAGTGGTGCTTATTTGAAAGGCAGAAACAGGCAGGCTGGGATGCCCGGAGCACCGGTCAGGGGTCTCTCCGGCTGCTCTCTTCTGCTGAGAGTGCCTCATAGAAAATGTTCCGTCTGTCTGGGATGTAAGCAGTCCTGGGAGTGGGCAGGTCTCCGCGGAAGGTGAGTCAGAAGACCCTGGATATATGTGAGTTGCTCTCAAGTGGCGGGCAAACAGGAACCTCCTGCTCTGCTGATTCTTTTGTGAAGGTGTTTTCTGTTTGTGTCTTCAGCGGAAACTTGCCCCATTTTTTATGACGTCTTTTTTGCGGTGGCCAATGGAAATGAATTACTGTTGGACTTGTCCCTCACAAAAGTCAATGCTACTGAACCAGAGAGAACAGCCATGAAAAAAATCCAGGATTGCTACGTGGAGAACGGACTCATATCCAGGGTCTTGGATGGACTAGTCATGGTAATTTCCTATCCTTCCCCGCCTCCCCAACCTTCACGTTGCGCGTGCAGCATATTGTAATATTCCACATACAGACCATGCAGTCAGGGGCTAATGGCAGGTAAGAGCTATAAACAATCGAGCACATAAACCTTTGCTCCGCGCTCTACAGCACATAGAATACGCAACCTCACGCCATGTGCACACCCAGCCTGTTCTTCTACCACACGTGTCCCTTGTGTGCGAATTACCTTACGCACAGTTGGAAAATAGGGGACTAATATCGGTGTGGCATAGAAAGCGTGTTGACTCGTAGGATTTTTTTCTTTCTAGGTTAGGGGTGTCAGAATTGCAGGAGTAGGATTTTAGCCTTCCACAGGAAAGAGAAAGTTCTTCATTCAGCTCCTGCACATGTAGGAGCCTTGTCAGTTCTAGTTGAGGAATATTGAAACTAAGCACCTGCCCTCAGACTCTCTTCCCAGGAAGGGACTCCCTGGCTTTGGGAAGCTTCTGGTTTTTGGCTTCTGTTTTACTTCCCCTTGTGCCCACCTTGATGGCTGCTATTCCTTTGGTTCAGAGTCTCACTTCCTTCTGTATCAATTCAGGGTCTAAAGTCAGATTTCCACTCTGTTTGTTCTGGTGCCTGAGGCCCTCGAGGCAGCTCCTAGCTACGTGCAGCTGCACCCCAGGGCTGGTCAGTGTATTTCTGGTGAACTATCTTTTTCTGTTATTTTTCTTGTTGCACAGTTAGGTCGATTTTGGTTAGTCTGTCTCTTACCTCTACTTGCCGTTAAGTGCTGATTCTGTAAAATGAGAGCTTTGTGAAGAAGTGGAATTTCTTGCATGACTACGGGCACCCAGGGCACATGGGATTGTTCACAACACACACATACACATTCCATACATCCAGTACACCTGACAGATGAGTCTCAGGTGAGGGAGACATCGCATGGACCCAGACTCAGCTACCTTGCCCCTCACCCAGGCCCATCCCCATCGCGCCCTCCAGAATCTTCTCCTCTTCTTGCCTCCTCACTGGTTGTTCAGACTCCTCTGGCACAGGTGCGTGGGTGACGGGGGGGGGGGGGGGGGGCGTCTCCATCCTGGTCTGACTGATCGCGGCCCTCTCTCCAGAAATCGGTCTGTGGGCTAGAGGTTCTTGCTAGGGACGGAGCGGAATCACTGGGGATGAGGCATGAGGTGATCCTGGGGGAATGGATACGCTGCCATGCGCTCAGGTCTTCTGTCCCTCCTCGTCTTACTCTCTCCCAGATAGCCATCAACGAATATTGCATGGGTGAAGCAGTTCAGAACACCGTAGAAGATCTCAAGCTGAACACTTTGGGGAGA**TGA**ATCTTTGCCGCTGATGCCCCTTCTGAGCCCCATCCTCCTGTCCTGTTCTTTACACCTAAAGCTGGAATCCAGACACCTGTCCTCACCTAATTCACTCTCAATCCAGGCTGACTAGAATCTGCAG

3’

**Supplementary Figure 4.** In vitro validation of sgRNA candidates in the CH2 genome. The genomic DNA of skin fibroblasts from the WT cat was used to prepare the PCR products. The size of the PCR product from Con to C2-2 was 296 bp. The efficiency of the sgRNA candidates of CH2 (C2-1 and C2-2) was calculated using the ImageJ program^15^. In vitro validation is presented as cleavage percentage. The sizes of the cut bands of C2-1 were 185 and 111 bp. The sizes of the cut bands of C2-2 were 194 and 102 bp. Both C2-1 and C2-2 showed high efficiencies in cleavage percentages.

Abbreviations: PCR, polymerase chain reaction; CH2, Fel d 1 chain 2; sgRNA, single-guide RNA; WT, wild type; M, size marker, 100 bp plus ladder; Con, Control.

**
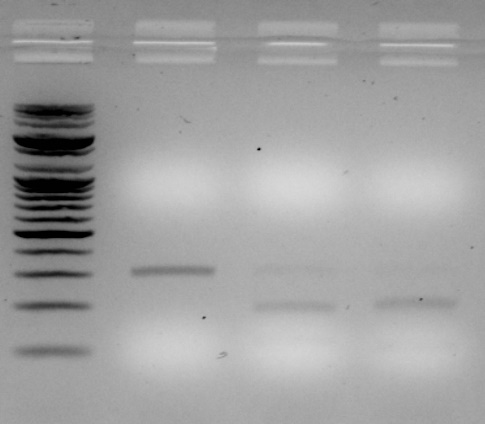
**

M Con C2-1 C2-2

500bp

Uncut bands

Cut bands

Cleavage % 0 81 85

**Supplementary**
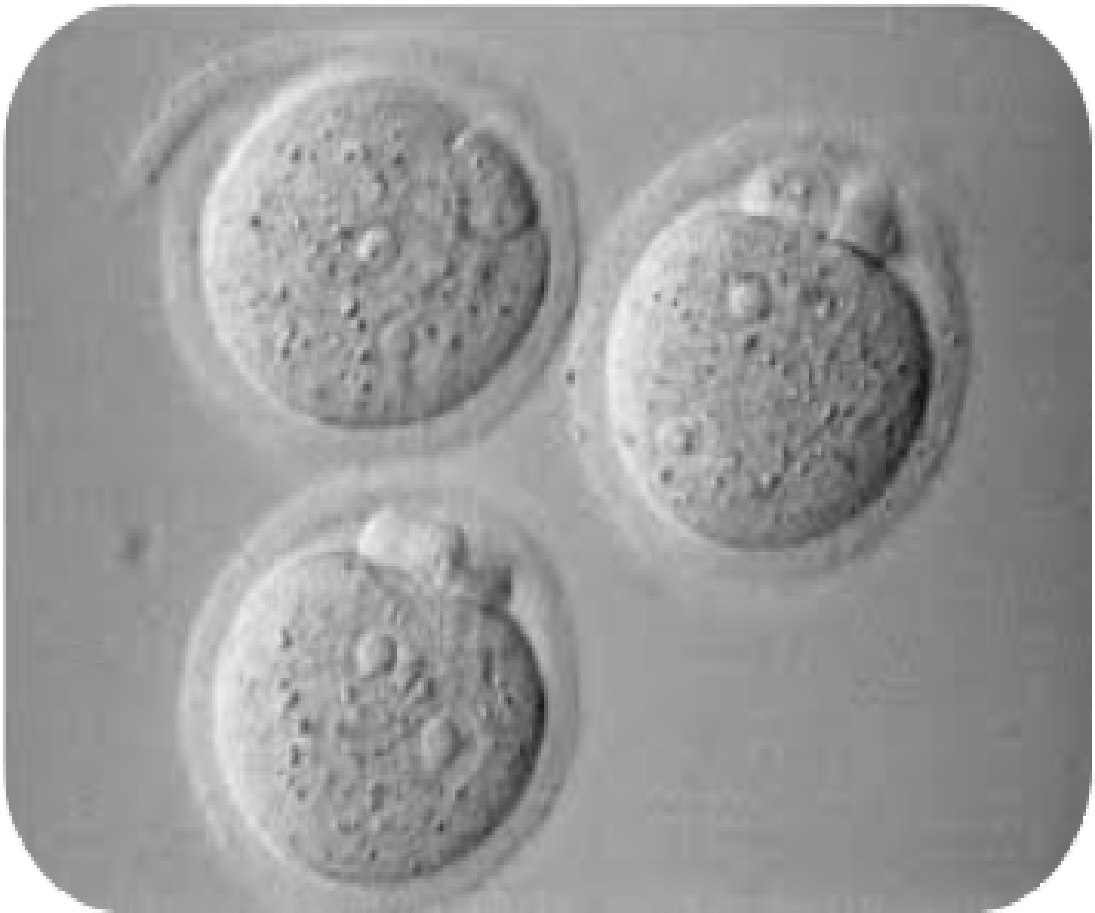
**Figure 5.** Comparison of one-cell stage embryos between cats and mice. (**a**) Cat one-cell stage embryos are filled with dark lipids, making it impossible to find the pronucleus. (**b**) Mouse one-cell stage embryos. The two pronuclei inside the mouse embryos are clearly shown.

**a b**

**Supplementary Figure 6.** The overall experimental process to create CH2 genome-edited cats. Oocytes were recovered via laparotomy.

Abbreviations: IVM, in vitro maturation; IVF, in vitro fertilization; IVC, in vitro culture.

Recovery of immature oocytes

Test in embryo development

Fertilization of oocytes and frozen sperm

Microinjection of gRNA/Cas9 into cytoplasm

Embryo implantation in oviduct

**IVM** (4 h)

4

**IVF**

(6 h)

**IVC**

(1 h)

**Supplementary Figure 7.** Generation of CH2 genome-edited founder cats using the CRISPR-Cas9 system. The pseudopregnant recipient female (mother) and two CH2 genome-edited founder kittens (male, KS-M-001, Heavy; female, KS-F-011, Haemi). Two CH2 genome-edited founder kittens were born after the microinjection of C2-1 and Cas9 mRNA and embryo transfer.


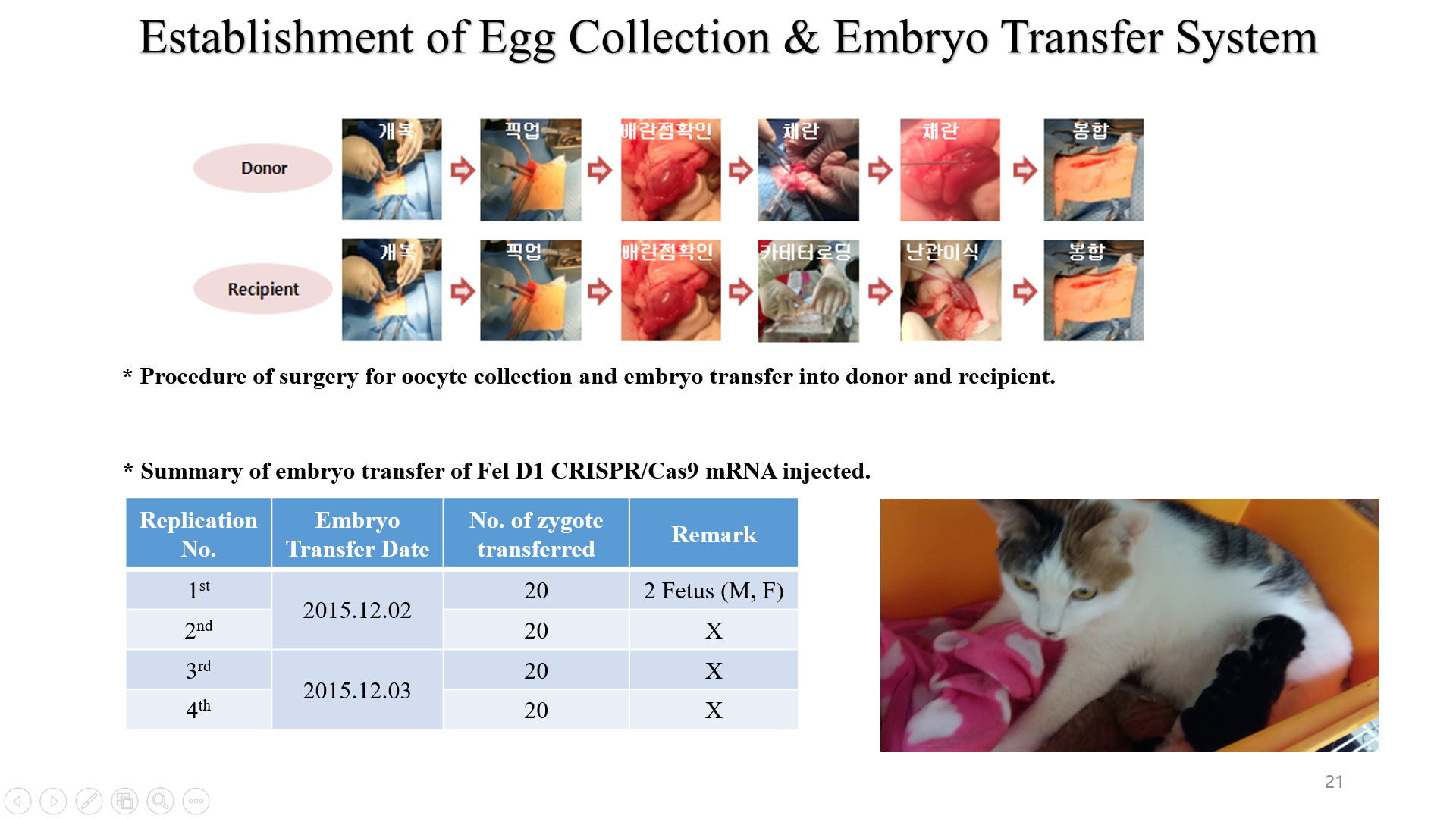


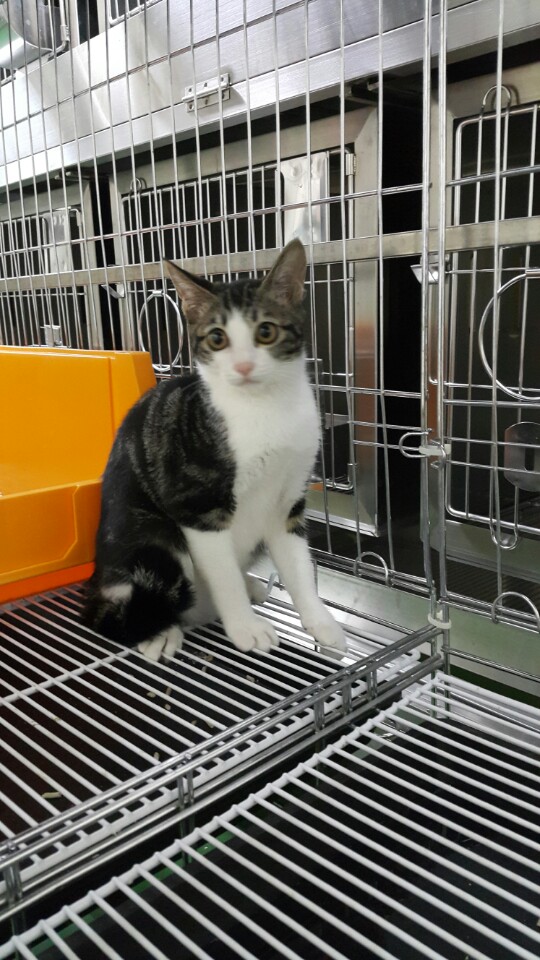

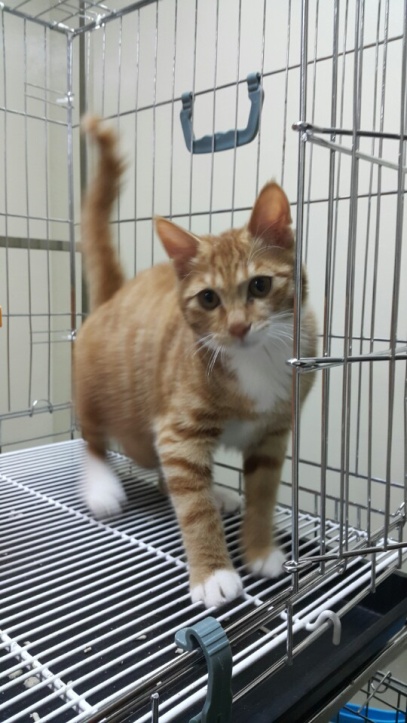


Heavy Haemi

**Supplementary Figure 8.** T7E1 assay of the C2-1 target region in two CH2 genome-edited founder cats (Heavy and Haemi). The WT cat only showed an uncut band (296 bp). “Heavy” and “Haemi” showed the cut bands (red arrow) as well as the uncut band (black arrow, 296 bp).

Abbreviations: PCR, polymerase chain reaction; CH2, Fel d 1 chain 2; sgRNA, single-guide RNA; WT, wild type; M, size marker, 100 bp plus ladder.

M WT Heavy Haemi

**
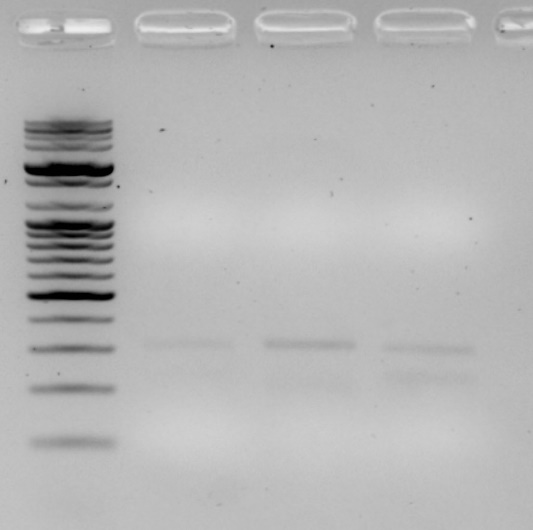
**

296 bp

500 bp

Cut band

**Supplementary Figure 9.** The Pedigree of the CH2 genome-edited cats. The two CH2 genome-edited founder cats (Heavy and Haemi) were mated, and six new kittens were born (M-003, M-004, M-005, M-006, M-007, and Alsik). Among these kittens, “Alsik” is a CH2 homozygous genome-edited cat.

KS-M-001 KS-F-011


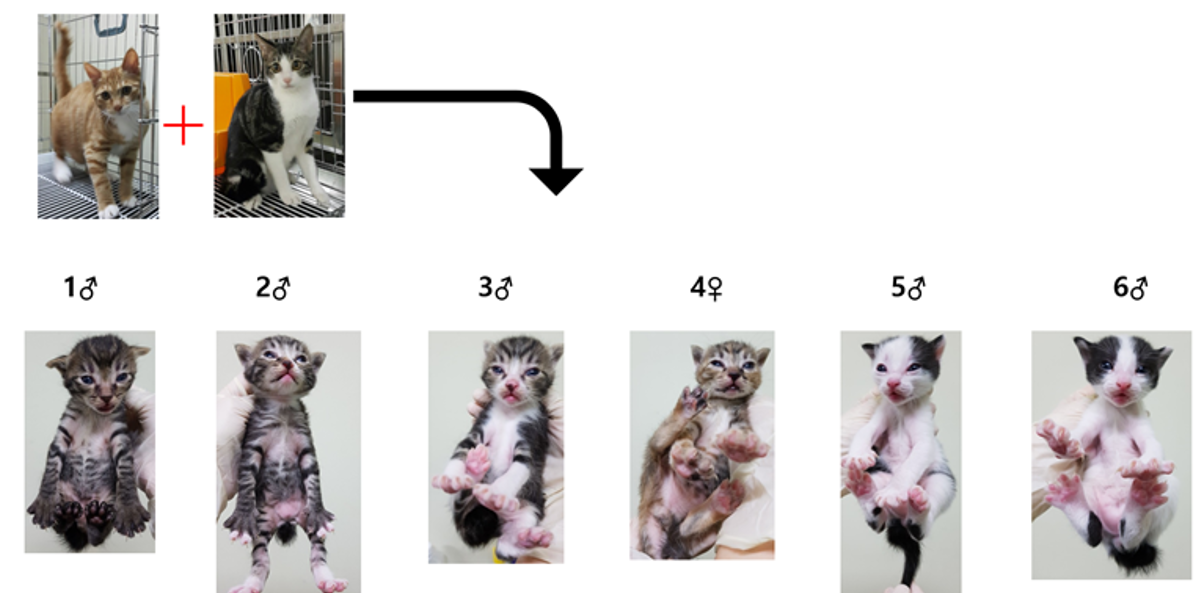
 Heavy Haemi

M-003 M-004 M-005 M-006 M-007 M-008

CH2^+/-^ CH2^+/-^ CH2^+/-^  CH2^-/-^

Alsik


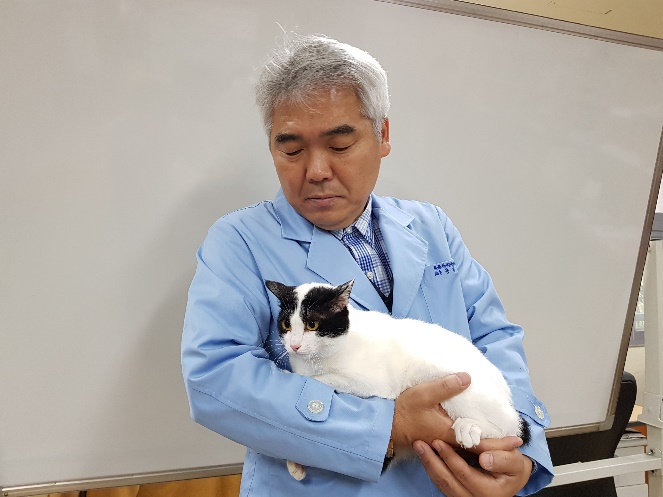


**Supplementary Figure 10.** T7E1 assay of the C2-1 target region in a CH2 homozygous genome-edited cat (Alsik). The WT cat and “Alsik” showed only an uncut band (296 bp). This implies that “Alsik” is a CH2 homozygous genome-edited cat. Sanger sequencing of these bands is shown in Fig. 1.

Abbreviations: PCR, polymerase chain reaction; CH2, Fel d 1 chain 2; sgRNA, single-guide RNA; WT, wild type; M, 100 bp plus ladder, size marker.

**
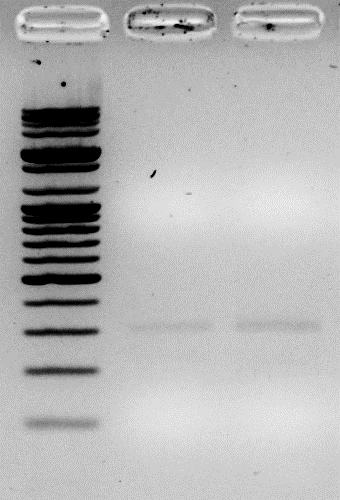
**

M WT Alsik

500 bp

296 bp

**Supplementary Figure 11.** PCR amplification of off-target analysis on a CH2 homozygous genome-edited cat (Alsik). (**a**) PCR band for CH2OTC1. (**b**) PCR band for CH2OTC2. (**c**) PCR band for CH2OTC3. (**d**) PCR band for CH2OTC4. The PCR bands (**a-d**) flanking each predicted off-target site in the CH2 homozygous genome-edited cat (Alsik) were visualized using agarose gel electrophoresis. No nonspecific band was observed for any PCR reaction. CH2OTC1, CH2OTC2, CH2OTC3, and CH2OTC4 are candidates for off-target analyses.

**a b c d**

**
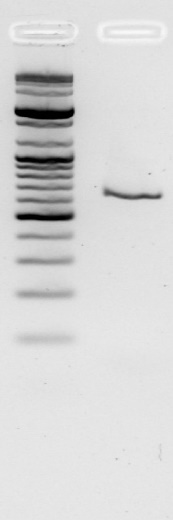

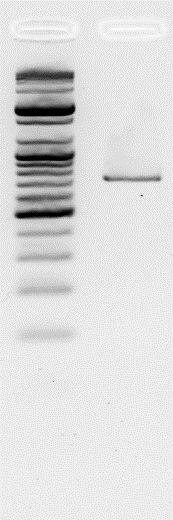

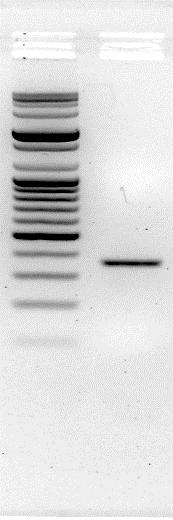

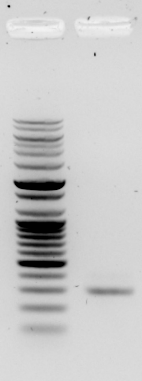
**

727 bp

650 bp

353 bp

283 bp

**Supplementary Figure 12.** Sanger sequencing of PCR amplification for off-target analysis of the CH2 homozygous genome-edited cat (Alsik). Four off-target candidates (**a-d**) for the CH2 homozygous genome-edited cat (Alsik) with representative sequencing are shown. Candidate sequences are shown in the red box. None of the sequencing results showed off-target mutations. CH2OTC1 (**a**) CH2OTC2 (**b**) CH2OTC3 (**c**) and CH2OTC4 (**d**) were candidates for off-target analysis.

**a**


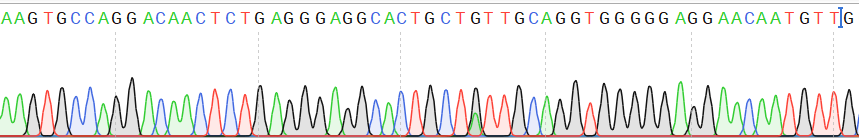


**b**


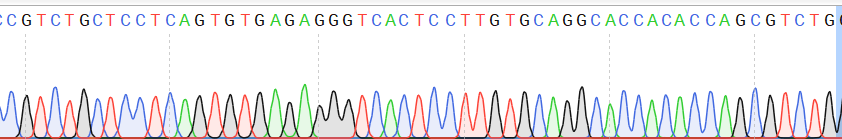


**c**


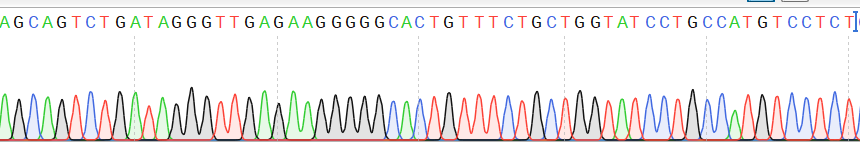


**d**

**
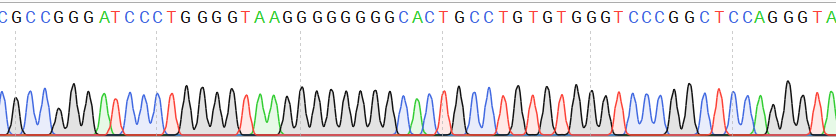
**

**Supplementary Tables**

**Supplementary Table 1.** sgRNA candidates in the CH2 genome. The bold red nucleotides represent the PAM sequences.

Abbreviations: CH2, Fel d 1 chain 2; sgRNA, single-guide RNA; PAM, protospacer adjacent motif.

| C2-1; 5’ GAGGGGGGCACTGCTTGTGC**TGG** 3’ |
| --- |
| C2-2; 5’ ACTGCTTGTGCTGGCATTGC**TGG** 3’ |

**Supplementary Table 2.** PCR primers used for C2-1 mRNA synthesis. The underlined nucleotides indicate the T7 promoter sequence and the bold underlined nucleotides indicate the start of the transcription site.

Abbreviations: PCR, polymerase chain reaction; F, forward; R, reverse.

| Name | Sequence (5′ -> 3′) |
| --- | --- |
| C2-1 F | TAATACGACTCACTATA**G**GGAGGGGGGCACTGCTTGTGCTGG |
| C2-1 R | AAAAGCACCGACTCGGTGCC |
| Cas9 F | TAATACGACTCACTATA**G**GGAGAATGGACTATAAGGACCACGAC |
| Cas9 R | GCGAGCTCTAGGAATTCTTAC |

**Supplementary Table 3.** Superovulation and synchronization of donor and recipient queens. Abbreviations: PMSG, pregnant mare serum gonadotropin; hCG, human chorionic gonadotropin.

| Types of Cats | Day 1 | Day 5 | Day 6 |
| --- | --- | --- | --- |
| Donor | PMSG | hCG | (8:00 AM) Aspiration & collection of the cat oocytes |
| Recipient | PMSG | hCG | (8:00 PM) Implantation of the injected embryos |

**Supplementary Table 4.** Experiment status of cytoplasmic microinjection of C2-1 and Cas9 mRNA into cat one-cell stage embryos and embryo transfer. In 1^st^ embryo transfer experiment, one female recipient was the only one to give birth. If there is no reason to be concerned during the embryo transfer, the other three recipient females do not become pregnant despite having been fertilized.

Abbreviations: CH, chain; sgRNA, single-guide RNA; M, male; F, female; N/A, non-applicable.

| Replication number | Number of embryos transferred per recipient | Pups | Indels |
| --- | --- | --- | --- |
| 1^st^ | 20 | 2 Kittens  (KS-M-001, M / KS-F-011, F) | 2/2 |
| 2^nd^ | 20 | No birth | N/A |
| 3^rd^ | 20 | No birth | N/A |
| 4^th^ | 20 | No birth | N/A |

**Supplementary Table 5.** The primer for CH2 genotyping. Abbreviation: CH2, Fel d 1 chain 2.

| Genes | | Sequence (5’ -> 3’) | Size (bp) |
| --- | --- | --- | --- |
| CH2 | PCR | Forward primer; TCTTAACAGCCAAGGGTTGAG | 296 |
|  |  | Reverse primer; GGAGGTGAAGGAGTGAGATAGA |  |

**Supplementary Table 6.** Primers used for microsatellite analysis. The 5’ end of each forward primer was labeled with 6-FAM.

| Candidates | Primers (5′ -> 3′) |
| --- | --- |
| FCA078 | Forward primer; TGAACTGAAGTCAGATGCTTAACC |
|  | Reverse primer; CGGAATCAGCTATTTTTACGG |
| FCA170 | Forward primer; CAAGGCGTTTGGTATTTTGG |
|  | Reverse primer; TTTACAGTCTCCCTCCTGATGC |
| FCA201 | Forward primer; TCTGCAGGACCAGTCAGATG |
|  | Reverse primer; AGCATACACAAATTGATGCTGG |
| FCA224 | Forward primer; CTGGGTGCTGACAGCATAGA |
|  | Reverse primer; TGCCAGAGTTGTATGAAAGGG |
| FCA229 | Forward primer; CAAACTGACAAGCTTAGAGGGC |
|  | Reverse primer; GCAGAAGTCCAATCTCAAAGTC |
| FCA290 | Forward primer; GTAAGGTCCCTGCCACACAT |
|  | Reverse primer; GTATCTGCCCATTTGAGAAAGG |
| FCA304 | Forward primer; TCATTGGCTACCACAAAGTAGG |
|  | Reverse primer; CTGCATGCCATTGGGTAAC |
| FCA305 | Forward primer; GGGTATTTACCCAAGAACATGG |
|  | Reverse primer; TGTTCATACACATACCTGCGC |
| FCA441 | Forward primer; ATCGGTAGGTAGGTAGATATAG |
|  | Reverse primer; GCTTGCTTCAAAATTTTCAC |

**Supplementary Table 7.** Microsatellite analysis of the CH2 homozygous genome-edited cat (Alsik) and the cloned cat (Alsik C).

| Microsatellite | PCR product range (bp) | WT (bp) | M-008 (bp) | KS-M-004 (bp) |
| --- | --- | --- | --- | --- |
| FCA078 | 182–205 | 186/192 | 187/201 | 187/201 |
| FCA170 | 103–117 | 110/116 | 110/116 | 110/116 |
| FCA201 | 135–158 | 138/155 | 138/151 | 138/151 |
| FCA224 | 154–175 | 157/172 | 159/159 | 159/159 |
| FCA229 | 160–172 | 168/170 | 166/168 | 166/168 |
| FCA290 | 206–220 | 212/212 | 216/216 | 216/216 |
| FCA304 | 100–115 | 112/112 | 108/108 | 108/108 |
| FCA305 | 185–201 | 194/194 | 197/197 | 197/197 |
| FCA441 | 153–162 | 155/159 | 155/159 | 155/159 |

**Supplementary Table 8.** The production level of Fel d 1 in CH2 genome-edited cats. (**a**) Fel d 1 level in the saliva of CH2 genome-edited male cats before- after washing. *Significantly different compared to WT male (*P* < 0.05). **Significantly different compared to WT male (*P* < 0.05). ***Significantly different compared to “Heavy” (*P* < 0.05). (**b**) Fel d 1 level in the saliva of CH2 genome-edited queens before- and after washing. *Significantly different compared to WT female (*P* < 0.05). **Significantly different compared to WT female (*P* < 0.05). (**c**) Fel d 1 level in the fur of CH2 genome-edited male cats before- and after washing. *Significantly different compared to WT male (*P* < 0.05). **Significantly different compared to WT male (*P* < 0.05). ***Significantly different compared to “Heavy” (*P* < 0.05). (**d**) Fel d 1 level in the fur of CH2 genome-edited queens before- and after washing. *Significantly different compared to WT female (*P* < 0.05). **Significantly different compared to WT female (*P* < 0.05). ELISA was used to analyze Fel d 1 production levels, which were expressed as mean standard deviation. The graphs for each value are shown in Fig. 2. Abbreviations: WT, wild type; W, washing; W-1, the day before washing; W+1, the day after washing; W+4, day 4 after washing; W+7; day 7 after washing.

| Days | WT Male (µg/ml) | Heavy (µg/ml) | Alsik (µg/ml) |
| --- | --- | --- | --- |
| W-1 | 16.11 ± 0.18 | 12.20 ± 0.81^*^ | 5.86 ± 0.33^**, ***^ |
| W+1 | 17.61 ± 0.97 | 6.77 ± 0.54^**^ | 0.31 ± 0.04^**, ***^ |
| W+4 | 18.00 ± 1.81 | 12.70 ± 2.12^*^ | 2.04 ± 0.36^**, ***^ |
| W+7 | 10.69 ± 3.55 | 9.21 ± 1.17 | 0.15 ± 0.02^**, ***^ |

**a**

| Days | WT Female (µg/ml) | Haemi (µg/ml) |
| --- | --- | --- |
| W-1 | 16.80 ± 2.63 | 6.35 ± 0.99^*^ |
| W+1 | 13.49 ± 0.47 | 0.33 ± 0.00^**^ |
| W+4 | 17.06 ± 0.59 | 1.52 ± 0.13^**^ |
| W+7 | 11.81 ± 0.84 | 1.51 ± 0.10^**^ |

**b**

| Days | WT Male (µg/g) | Heavy (µg/g) | Alsik (µg/g) |
| --- | --- | --- | --- |
| W-1 | 13.60 ± 1.47 | 11.73 ± 0.25 | 3.75 ± 0.08^**, ***^ |
| W+1 | 7.91 ± 0.35 | 3.10 ± 0.39^**^ | 0.71 ± 0.15^**, ***^ |
| W+4 | 10.38 ± 0.43 | 6.42 ± 0.29^*^ | 0.24 ± 0.11^**, ***^ |
| W+7 | 13.06 ± 4.03 | 8.46 ± 1.06 | 0.10 ± 0.01^**, ***^ |

**c**

| Days | WT Female (µg/g) | Haemi (µg/g) |
| --- | --- | --- |
| W-1 | 7.03 ± 0.65 | 5.92 ± 0.16 |
| W+1 | 4.73 ± 0.05 | 1.51 ± 0.16^**^ |
| W+4 | 5.43 ± 0.65 | 1.86 ± 0.18^*^ |
| W+7 | 6.05 ± 0.63 | 1.34 ± 0.16^**^ |

**d**

**Supplementary Table 9.** Candidates for off-target analysis of the CH2 homozygous genome-edited cat (Alsik). Sequences of the four predicted off-target sites and each primer are displayed here. The blue highlights indicate mismatch sequences. The red highlights indicate PAM sequences.

Abbreviation: PAMs, protospacer adjacent motifs; CH2, Fel d 1 chain 2

| Candidates  (PCR product size) | Name | Sequence (5′ -> 3′) | Chromosome Location |
| --- | --- | --- | --- |
| CH2OTC1  (353 bp) | Target sequence | GAGGGaGGCACTGCTatTGCAGG | Chromosome A1 |
|  | Forward primer | CTCAAATGGTGTGGCAAGTTC |  |
|  | Reverse primer | TGGAGCAGCCTGATACAAAG |  |
| CH2OTC2  (727 bp) | Target sequence | GAGaGGGtCACTcCTTGTGCAGG | Chromosome B3 |
|  | Forward primer | TCTTCCACGGCACCAATAAA |  |
|  | Reverse primer | GTTAATGACCCTCCTCGCATAG |  |
| CH2OTC3  (650 bp) | Target sequence | GAaGGGGGCACTGtTTcTGCTGG | Chromosome C2 |
|  | Forward primer | TTTCTGTGGGCCTTGGATATAG |  |
|  | Reverse primer | CATCAACCATTACGCACCATTC |  |
| CH2OTC4  (283 bp) | Target sequence | GgGGGGGGCACTGCcTGTGtGGG | Chromosome C2 |
|  | Forward primer | GCGAATTTCTTTGTGTCGGGT |  |
|  | Reverse primer | GGATACCGAAGGCAAAGGGG |  |

| PCR Conditions | Denature | Anneal | Extend | Repeat |
| --- | --- | --- | --- | --- |
|  | 94°C for 30 s | 62°C for 30 s | 72°C for 1 min | 30 cycles |
